# Novel TEM Microscopy and Electron Diffraction Techniques to Characterize Cultural Heritage Materials: From Ancient Greek Artefacts to Maya Mural Paintings

**DOI:** 10.1155/2019/4870695

**Published:** 2019-05-22

**Authors:** Stavros Nicolopoulos, Partha P. Das, Alejandro Gómez Pérez, Nikolaos Zacharias, Samuel Tehuacanero Cuapa, Jesús Angel Arenas Alatorre, Enrico Mugnaioli, Mauro Gemmi, Edgar F. Rauch

**Affiliations:** ^1^NanoMEGAS Sprl, Blvd Edmond Machtens 79, B-1080 Brussels, Belgium; ^2^Electron Crystallography Solutions SL, Calle Orense 8, 28020 Madrid, Spain; ^3^Department of History, Archaeology and Cultural Resources Management, University of the Peloponnese, 24100 Kalamata, Greece; ^4^Instituto de Física, Circuito de la Investigación s/n, UNAM, Cd. Universitaria, Coyoacán, 04510 México D.F., Mexico; ^5^Center for Nanotechnology Innovation@NEST, Istituto Italiano di Tecnologia, Piazza San Silvestro 12, 56127 Pisa, Italy; ^6^SIMaP, Grenoble INP CNRS UJF, BP 46, 38402 Saint-Martin-d'Hères Cedex, France

## Abstract

To understand in-depth material properties, manufacturing, and conservation in cultural heritage artefacts, there is a strong need for advanced characterization tools that enable analysis down to the nanometric scale. Transmission electron microscopy (TEM) and electron diffraction (ED) techniques, like 3D precession electron diffraction tomography and ASTAR phase/orientation mapping, are proposed to study cultural heritage materials at nanoscale. In this work, we show how electron crystallography in TEM helps to determine precise structural information and phase/orientation distribution of various pigments in cultural heritage materials from various historical periods like Greek amphorisks, Roman glass tesserae, and pre-Hispanic Maya mural paintings. Such TEM-based methods can be an alternative to synchrotron techniques and can allow distinguishing accurately different crystalline phases even in cases of identical or very close chemical compositions at the nanometric scale.

## 1. Introduction

The analytical examination of glasses, glazed artefacts, and pigments associated with cultural heritage activities is an important field in archaeometry science. The techniques that are involved are either totally non- or quasi-destructive aiming at providing information towards chemical fingerprinting (e.g., XRF (X-ray fluorescence), ICP-MS (inductively coupled mass spectrometry), EDS (energy-dispersive X-ray spectroscopy), EPMA (electron probe microanalysis), XRD (X-ray diffraction), and Raman spectroscopy). In all those techniques, resulting data either provide average information from the bulk of the sample or from local analyses on areas that are several tens of microns, missing therefore critical information when various nanoscale mineralogical phases may be involved.

For the structural characterization of materials at the nm scale, transmission electron microscope (TEM) electron diffraction techniques like ASTAR phase/orientation mapping [1, 2] and electron diffraction tomography (ADT) [3, 4] have shown very promising results for a wide range of materials like metals, alloys, ceramics, natural minerals, semiconductors, nanowires, organic natural and synthetic pigments, organic materials, and pharmaceutical compounds [5].

In the case where structural information of the studied material is known, phase and orientation mapping provides information about the distribution of various phases in the sample; in the case where structural information for a given sample is completely unknown, electron diffraction tomography (ADT) in combination with chemical analysis helps precise structural characterization, providing precise information about individual phases of an object of interest. The development of TEM-related analytical tools like electron energy loss spectroscopy (EELS), high-resolution imaging (HREM), energy-dispersive X-ray spectroscopy (EDS), 3D imaging, and electron diffraction tomography in addition to the wider access/availability TEM in various laboratories worldwide has made TEM an interesting alternative synchrotron-based technique. On the other hand, combination of advanced electron diffraction-based techniques (phase/orientation mapping and electron diffraction tomography) has been used in few cases to solve complex material science problems [6], where its application in archaeometry has only been reported very recently [7–10]. In previous work [8, 9], we have focused on the study with ADT of small crystallites used as pigments in colored Roman tesserae (green, yellow, white, and different shades of blue). The present work is focused on the study of various pigments from different historical periods (Ancient Greek/Roman, pre-Hispanic) and different types of artefacts (amphorisks, glass tesserae, and Mayan mural paintings) using a combination of ADT and ASTAR orientation/phase mapping techniques in TEM to shed light in the various materials used/present in the artefacts. In this current article, our previous work on pigment characterization in yellow glass Roman tesserae [8] has also been summarized.

## 2. Materials and Methods

### 2.1. Sample Preparation

A small piece of Greek amphorisks was polished to a thin lamella a few millimeters wide, which was subsequently treated by ion milling to obtain electron beam transparent areas.

Electron beam transparent lamellae of Roman *tesserae* (4 × 4 *μ*m size and approximately 100 nm thick) were prepared by focused ion beam (FIB) using the FEI Helios NanoLab 600 DualBeam FIB, at LMA Zaragoza (Spain). The FIB protocol for lamella preparation was described in detail elsewhere [8]. The sample was lifted out from FIB and attached to specific TEM grids for further TEM analysis.

Pre-Hispanic pigment samples were prepared by grinding the powder in a mortar and then transferred by drop suspension on a carbon-coated copper TEM grid.

### 2.2. Analytical Techniques and Instrumentation

For TEM and STEM-EDS (scanning transmission electron microscopy–energy-dispersive spectroscopy) observations, a TEM JEOL-2100F (FEG, 200 kV) and a LIBRA 120 (LaB_6_, 120 kV), both equipped with a DigiSTAR precession system and ASTAR phase/orientation mapping system (NanoMEGAS Sprl, Belgium) [11], were used. The chemical composition from the polished thin cross sections were obtained by EMPA (electron microprobe analyzer), using JEOL JXA-8230 at the Centres Científicsi Tecnològics of the Universitat de Barcelona, Spain. It is useful to note that the STEM-EDS spatial resolution is much superior to that of EMPA (3-5 nm vs. 1 micron) where the STEM-EDS energy resolution is inferior compared to that of EMPA (5-20 eV vs. 130-150 eV). Therefore, in the case of chemical composition analysis of samples at the nm scale, a combination of both techniques is essential, especially when there is a possibility of overlapping chemical signals.

**Figure 1:**
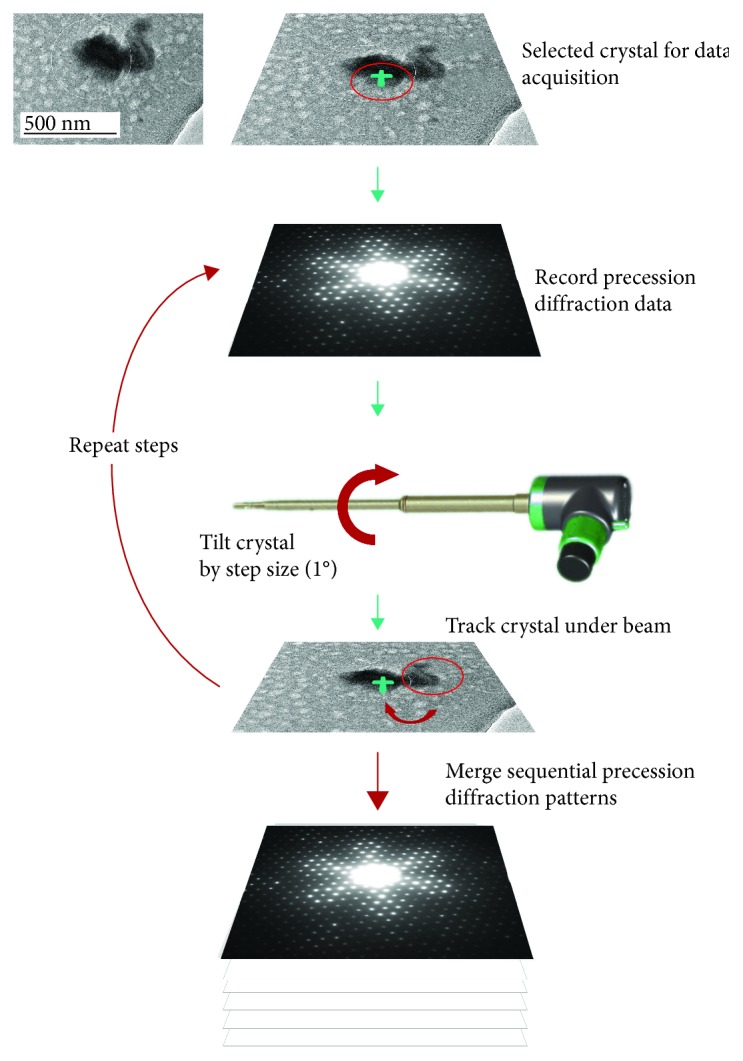
Electron diffraction tomography (ADT): diffraction patterns are collected by sequential tilting of crystal, adapted from previous work [9].

Electron diffraction tomography (ADT) is a recently developed TEM-based technique that, by collecting 3D electron diffraction (ED) data from single nanocrystals, enables to determine ab initio and refine their atomic structure (Figure 1) [3–5, 9].

ADT consists of sampling the reciprocal space of the target crystal in small tilt steps (usually of 1°), without the need of any prior knowledge of its crystal structure or crystal orientation. The reconstructed diffraction volume contains all reflections present in the sampled portion of the reciprocal space. The ADT technique can be performed in any TEM using a standard single tilt, tomographic holder, or cryo-holder. A range of tilt from -50° to +50° along the goniometer axis is normally available for most TEM models [4]. During a typical ADT experiment, the crystal position is tracked by TEM or STEM (scanning-transmission electron microscopy) imaging; the latter allows working with small electron doses, where it is possible to obtain good-quality and complete ED data sets also from beam-sensitive materials (like porous materials, hydrated materials, organics, and pharmaceuticals) [12, 13].

Precession electron diffraction (PED) is based on the precession of the electron beam around a cone surface. The typical precession aperture semi-angle is 1-3°. In the last 15 years, PED emerged as a technique suitable for crystal structure determination of various nanomaterials, as it significantly reduces the dynamic contribution in ED reflection intensities [11, 14, 15]. The combination of PED and ADT significantly improves reflection intensity integration, making correct crystal symmetry (space group) identification easier. Statistics on PED intensities may enable to distinguish between crystals having a similar unit cell (e.g., as close as 1-2% in cell lengths and 1-2° in cell angles) but different crystal symmetries. Moreover, the combination of PED and ADT significantly increases the chance to get an ab initio structure solution via direct methods. In our work, ADT and PED analyses were performed in a Zeiss Libra 120 microscope working at 120 kV, at the Istituto Italiano di Tecnologia-CNI@NEST Pisa (Italy), and with a JEOL 2100F working at 200 kV, at SIMaP, Grenoble (France).

**Figure 2:**
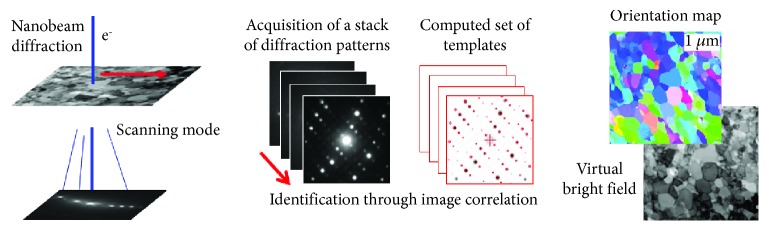
Setup for the ASTAR technique: electron diffraction patterns are collected while the beam is scanned over the sample. All patterns are compared to a set of precalculated templates to recognize the local orientation. Typical outputs are orientation/phase maps as well as virtual bright- or dark-field images.

Concerning transmission electron microscopy (TEM), there is an increasing interest for the so-called scanning precession electron diffraction (SPED) mode, commercially known as ASTAR. In this approach, 2D diffraction patterns are systematically acquired with fast cameras while the focused probe is scanning the area of interest (Figure 2). This enables the reconstruction of 2D maps highlighting different crystalline phases, crystallographic orientation and/or local stress fields, and presence of amorphous areas in the matrix at the nanoscale with spatial resolution up to 1-3 nm in typical areas of several *μ*m^2^ [1]. A small convergence angle is required because it produces spot-type diffraction patterns that may be automatically indexed with dedicated commercial software. For enhanced crystallographic orientation and phase indexing, the patterns are sorted by a specific strategy that uses image correlations; templates (i.e., theoretical diffraction patterns) are precalculated for all expected phases and all possible orientations and are systematically compared to the successively acquired patterns. The degree of matching between every template and the current diffraction pattern is estimated through a correlation index whose highest value corresponds to the best-fitting template and consequently to the probable orientation and phase. Precession is used to decrease dynamical effects and greatly increases the matching reliability especially low-symmetry phases as in the present work. Phases and orientation analysis presented in the current work were performed with a JEOL 2100F at SIMaP, Grenoble (France).

## 3. Results and Discussion

### 3.1. Blue Pigment TEM Characterization in Ancient Greek Amphorisk

**Figure 3:**
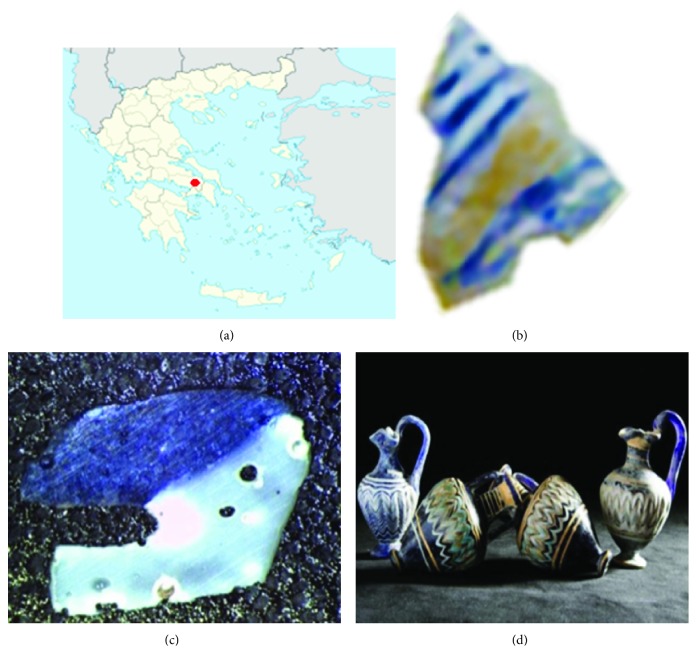
(a) Region of Thebes where glass amphorisks were found; (b) blue-colored glass fragment; (c) optical microscope image of the thinned slice; (d) typical ancient amphorisks.

**Figure 4:**
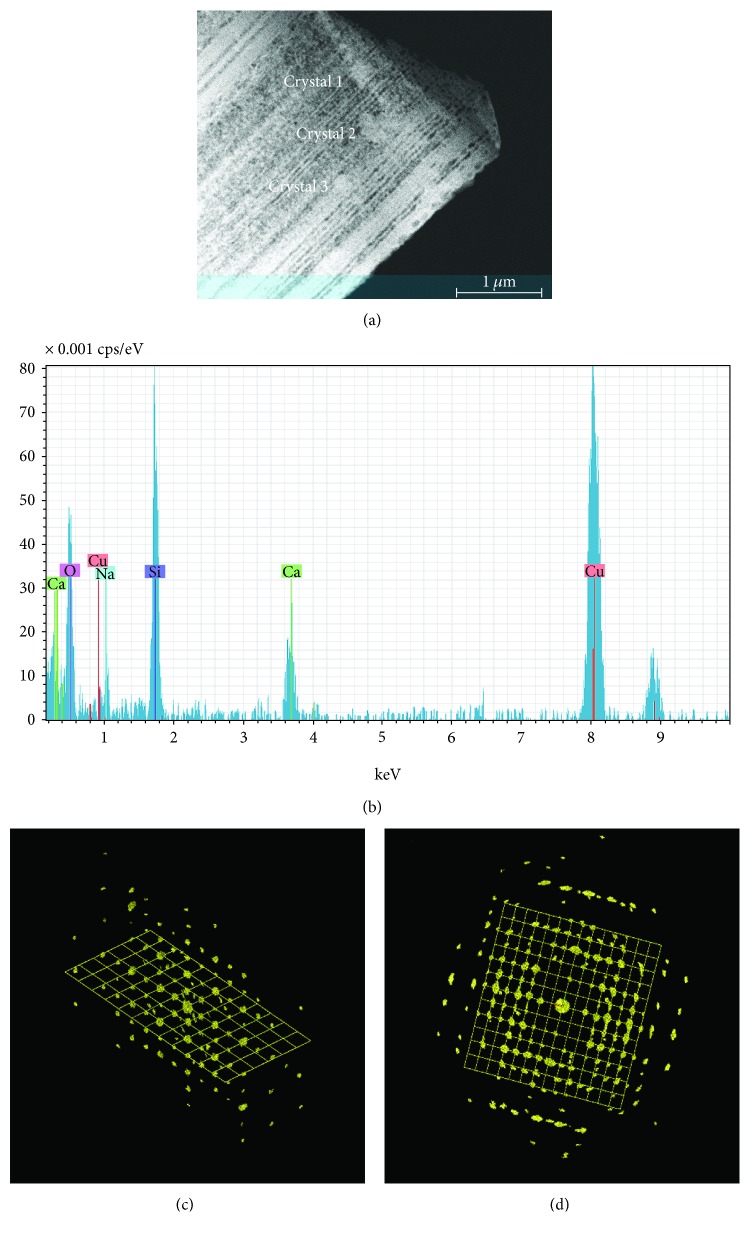
(a) Blue-colored glass TEM sample containing crystalline inclusions (marked as crystals 1-3) embedded in an amorphous glass matrix. (b) EDX measurement from one of the crystallites. ADT-reconstructed volumes of individual nanocrystalline precipitates with hexagonal *P*6/*mcc* symmetry, viewed along [001] (c) and along [110] (d) reciprocal space directions, respectively.

We applied the ADT technique for investigating the origin of the glazed blue color in a small pottery fragment, part of a bigger glass amphorisk of approximate dimensions 2 × 4 × 8 cm, which was found during a rescue excavation in the outskirts of Thebes (Greece) and dated to the Classical Period (late 6^th^–early 5^th^ century BC) (Figure 3). A very small part of the blue glazed fragment has been ion-thinned to electron transparency and prepared for TEM analysis.  Figure 4(a) shows the studied area, which contains crystalline inclusions of about 200 × 200 nm embedded in an amorphous glass matrix.

**Figure 5:**
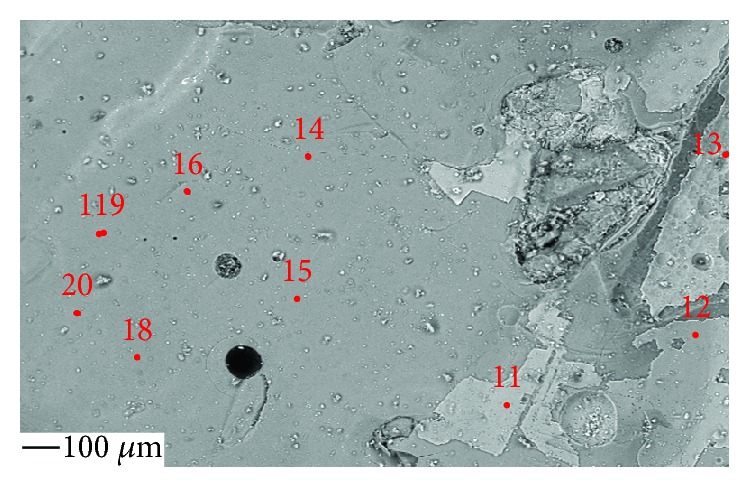
Polished glass cross section for EPMA measurement.

STEM-EDS analysis on both glass matrix and inclusions shows the presence of Si, O, Al, Na, Sb, and Ca (Figure 4(b)). EPMA analysis (Table 1 and Figure 5) confirms the presence of the previous elements as well as Cu and a small quantity of Ti and cobalt oxide (0.26 wt%). ADT analysis was made on 3 different crystalline inclusions from the glass matrix. The analysis of the 3D-reconstructed diffraction volume and reflection intensities (576 in total) show a hexagonal symmetry with the *P*6/*mcc* space group and unit cell *a* = *b* = 10.41 Å and *c* = 13.84 Å (Figures 4(c) and 4(d)).

The combination of unit cell, symmetry, and elements detected by EDX (Ca, Si, Cu, Na, and O) leads to the conclusion that such inclusions belong to the milarite-osumilite mineral type with general formula K_2_Ca_4_Al_2_Be_4_Si_24_O_6_ for milarite and (K,Na)(Fe^2+^,Mg)_2_(Al,Fe^3+^)_3_(Si,Al)_12_O_30_ for osumilite, respectively. Though natural milarite-osumilite does not contain Cu^2+^ and Co, Nguyen et al. [16] have reported the synthesis of several milarite-osumilite minerals with general formula A_x_M_3_M′_2_Si_12_O_30_ (A = Na, K, Rb; M = Mg, Zn, Fe^2+^, Cu^2+^, Li and M′ = Mg, Cu^2+^, and Fe^2+^).

Is interesting to note that the identical phase has been also discovered in a blue glazed steatite interface of a 1130 BC scarab of Egyptian origin found in Sardinia, Italy [17]. Cu was used in ancient Egypt to produce a typical blue color of faience and glass, and in our case CuO is present in the sample (0.18 wt% as found by EPMA); it is also possible that similar Egyptian glazing and color technologies were widely used in the Mediterranean area already in the 5th c. BC. The presence of CoO 0.26 wt% undoubtedly has a strong influence in the observed blue color of the glaze as is known that as little as 0.01% CoO is enough to produce a blue color in the glass [18, 19].

### 3.2. Characterization of Pigment in Yellow Glass Roman Tesserae

**Figure 6:**
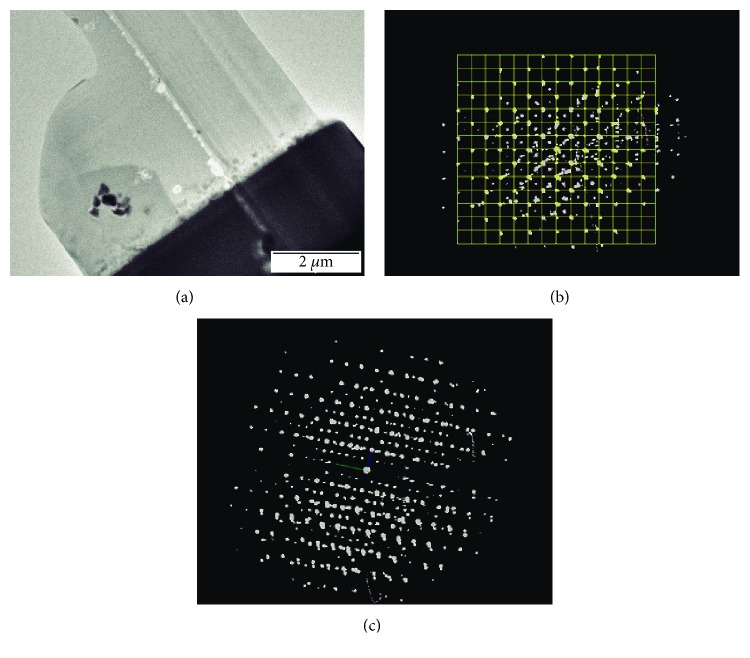
Yellow tesserae: (a) FIB thin slice containing several pigment/opacifier crystals. (b) 3D tomography reciprocal space projection of one of the studied crystallites along the [100] direction. (c) View of a 3D reciprocal space of the acquired data from a nanometer-size crystal.

In a previous work, we have analyzed FIB lamella samples from a yellow-colored glass tesserae [8], recovered in a large collection of Roman tesserae excavated in 2008 at the sanctuary of Isis and Serapis within the archeological site of Ancient Messene (Peloponnese, Greece) [20]. A TEM examination of the lamellae (Figure 6(a)) reveals the presence of small precipitates (200-400 nm) embedded in an amorphous glass matrix and containing Pb and Sb. Later on, we have extended our study by the ADT analysis of several embedded crystallites (Figures 6(b) and 6(c)). All the areas deliver a cubic unit cell with *a* = 10.53 Å and *Fd*-3m symmetry [8].

Based on ADT-PED reflection intensities and chemical information from STEM-EDS mapping, the crystal structure of the crystallites was solved by simulated annealing [21], leading to the determination of all Pb, Sb, and O atomic positions in the structure already reported in our previous work [8]. The so-determined Pb_2_Sb_2_O_7_ compound is indeed a well-known yellow color opacifier used already in ancient Mesopotamian and Egyptian civilizations [22–24].

### 3.3. New Pre-Hispanic Pigment Discovery and Full-Pigment TEM Characterization by Phase Mapping

**Figure 7:**
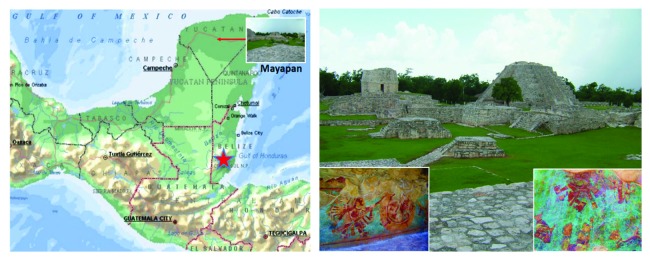
Archaeological place of Mayapan in southeast Mexico with Maya mural paintings.

**Figure 8:**
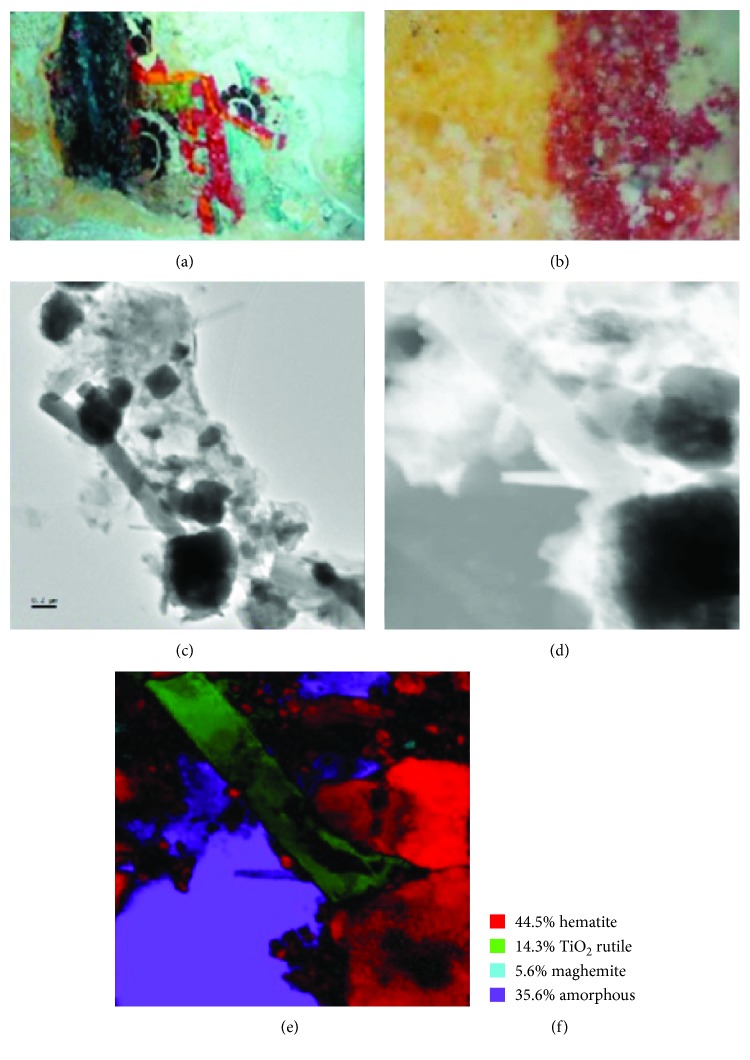
(a) Red sample from the mural of Round Temple. (b) Optical microscope view of the red pigment. (c) Area that was analyzed by TEM is shown. (d) TEM virtual bright field area where the analysis of phase mapping was done with ASTAR and is shown in (e). (f) Various phase percentages obtained with phase mapping are shown.

**Figure 9:**
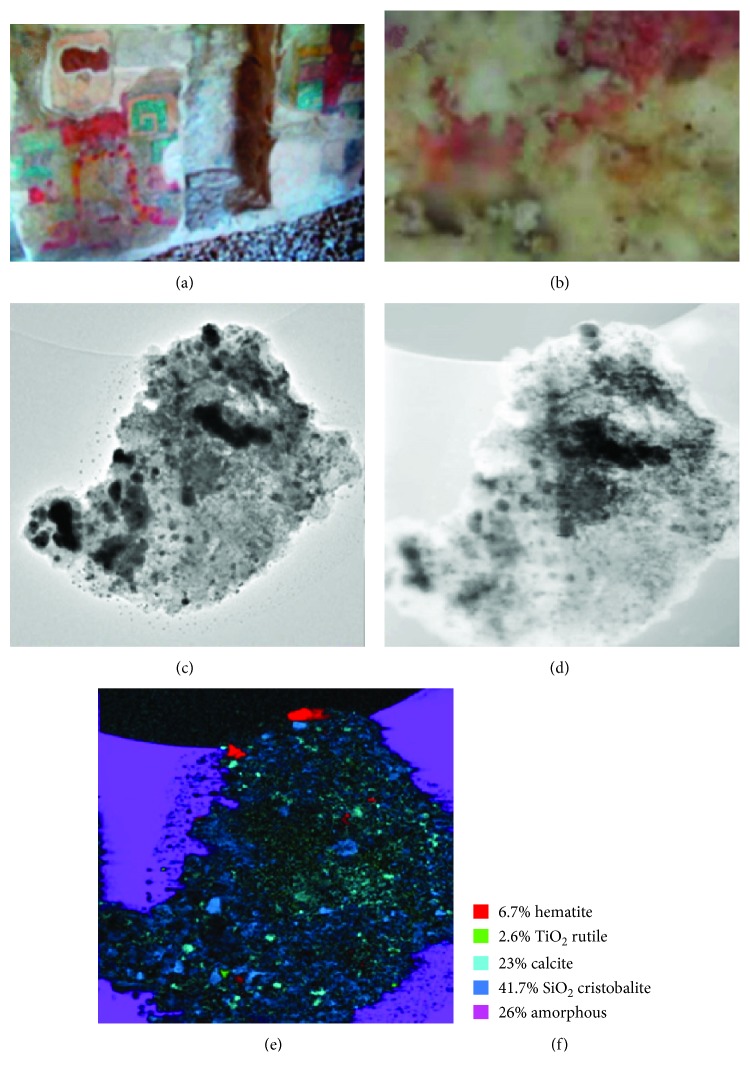
(a) Painted Niches Temple where the red sample was obtained to analyze it. (b) An optical microscope view of the red pigment is shown. (c) The area analyzed by TEM is shown. (d) TEM virtual bright field area where TEM phase mapping analysis was done and is shown in (e). (f) Various phase percentages obtained with phase mapping are shown.

**Figure 10:**
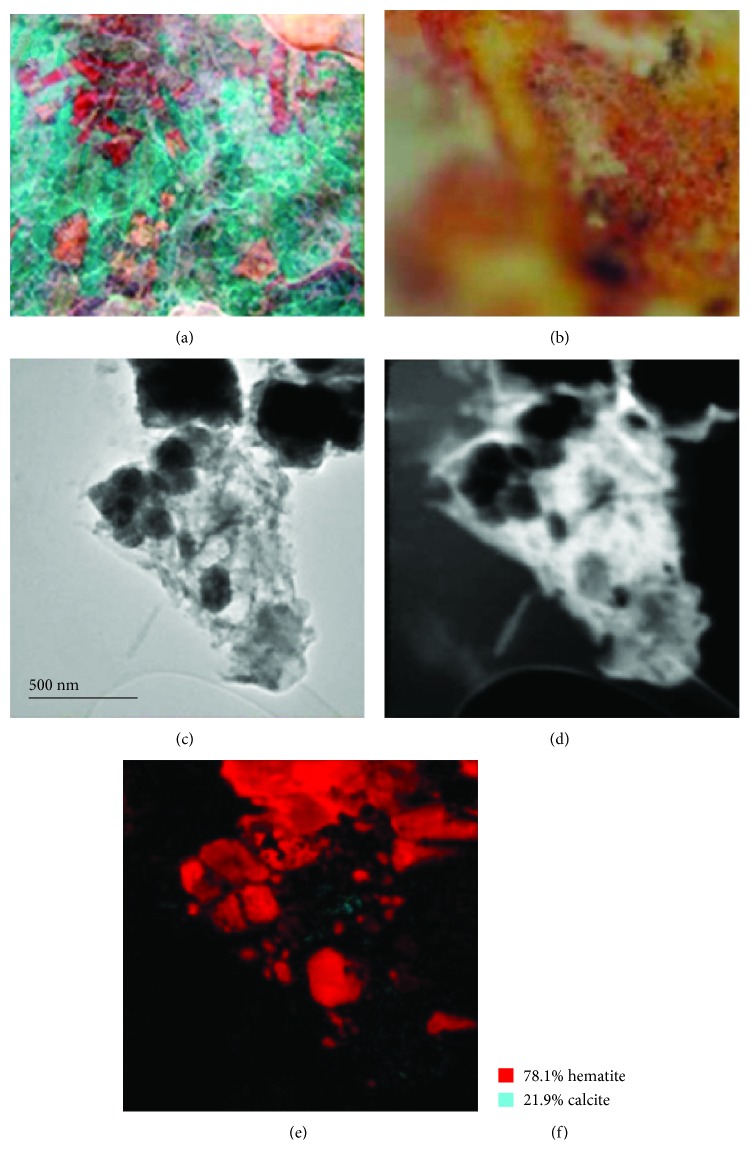
(a) Mural from Fisherman's Pyramid is shown. (b) An optical microscope view of the red pigment is shown. (c) Bright field TEM image. (d) TEM virtual bright field area where the analysis of TEM phase mapping was done and is shown in (e). (f) The phase percentage of hematite and calcite particles is shown.

**Figure 11:**
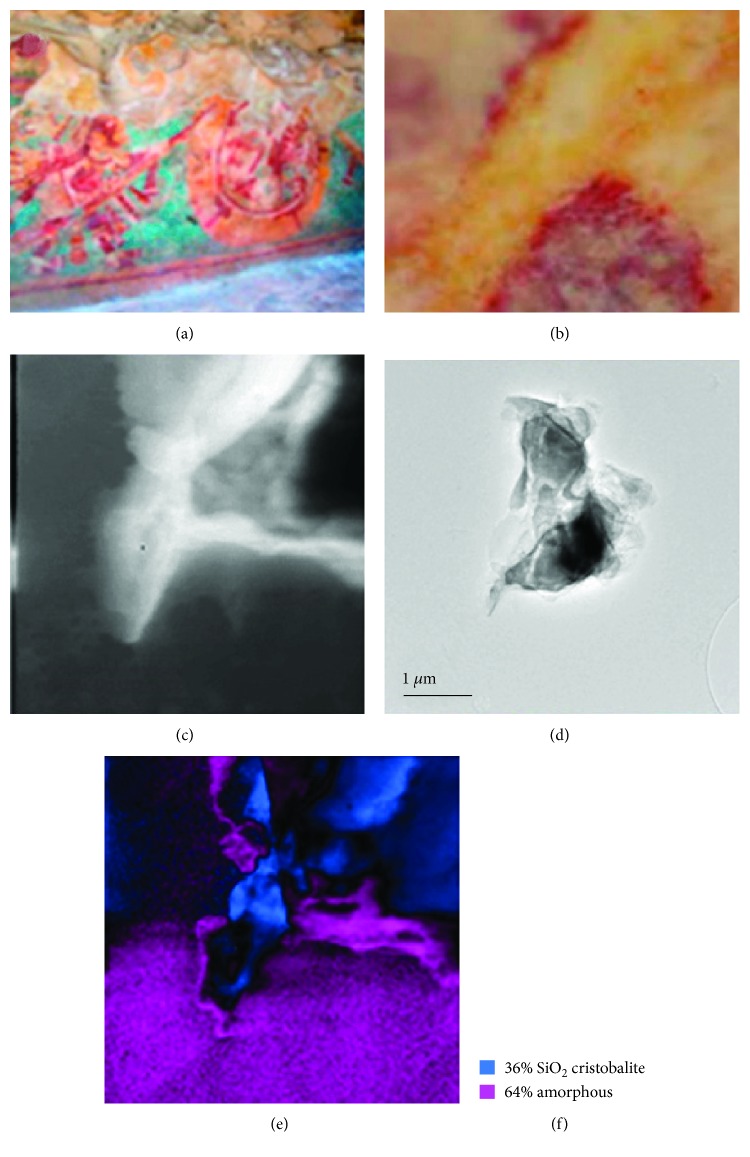
(a) The mural of the Solar Symbols Temple is shown. (b) Optical microscopy view of the analyzed area of the red pigment. (c) Bright field TEM image. (d) TEM virtual bright field area where the analysis of TEM phase mapping was done and is shown in (e). (f) The amount of crystalline cristobalite and amorphous carbon materials is shown in the Mayapan mural.

We have applied the TEM phase/orientation mapping technique (ASTAR) in the study of pre-Hispanic Maya pigments coming from the Mayapan site (Yucatan Peninsula, Mexico) (Figure 7). Several pigments with different colors have been analyzed (blue, green, and red) in order to understand the nature of the materials used for their preparation. Detailed phase mapping analysis by ASTAR phase mapping confirmed the presence of hematite (Fe_2_O_3_) as one of the major components used in the red pigments found in Round Temple, Painted Niche Temple, and Fisherman Temple (Figures 8–10); on the other hand, in Solar Symbols Temple the presence of crystalline cristobalite (Figure 11) was found. In addition, a small amount of nanocrystalline TiO_2_ (rutile) particles (previously undetectable by X-ray studies) has also been consistently detected using phase mapping in two of the studied red pigment samples (Figures 8 and 9). Rutile TiO_2_ impurities could come from the mineral products extracted from the red soil of the region; this is an important result, because it demonstrates the precision of the phase mapping technique to analyze pre-Hispanic pigments. What is very important is that, although rutile is a well-known white pigment, there is no previous evidence of its use in the Maya's culture or in any other ancient culture to our present knowledge [25].

### 3.4. Pigments of the Round Temple

**Figure 12:**
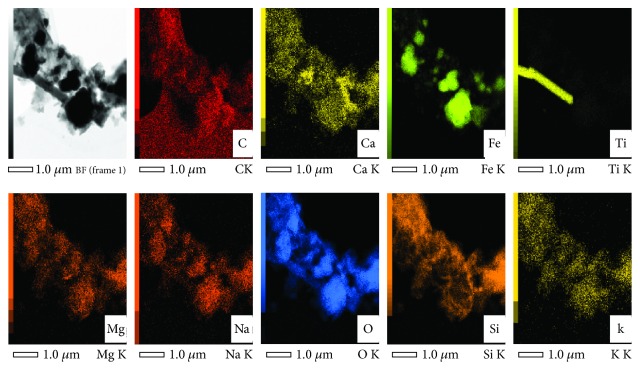
STEM-EDS elemental analysis of a selected area of the red pigment Round Temple Mayapan mural.

The red pigment that is next to yellow pigment (not presented in this work) was also analyzed. According to our knowledge, for Maya mural manufacture, first the yellow pigment was used, followed subsequently by the red pigment that was applied. In this sample, TiO_2_ impurities were also found and it was determined that they correspond to the rutile phase (Figures 8 and 12). Mainly, the hematite iron oxide's presence gives the red color to this pigment, but the maghemite iron oxide's presence (which was determined by using the TEM phase map) may also contribute to the red color.

### 3.5. Pigments of the Niches Temple

It was observed that the pigment is partially covered by particles of different shades, most likely the result of its interaction with the environment. In this case, using TEM phase mapping we also observe the presence of TiO_2_ particles (Figure 9) and SiO_2_ particles that probably come from the soil from where the hematite is extracted. Detected calcite particles possibly come from the support where the pigment is placed.

### 3.6. Pigments of the Fisherman's Pyramid

The Mayans used a paste of lime with a mixture of two or more vegetable gums from the barks of certain trees, which functioned as binders in the supports and in the pictorial layer to keep the particles of the pigments joined together, and to the support. In Figure 10, using TEM phase mapping, calcite particles are observed, indicating that possibly the hematite particles are attached to the calcite support.

### 3.7. Pigments of the Solar Symbols Temple

The presence of a noncrystalline material and the identification of carbon by TEM in this pigment (Figure 11) confirm the use of some organic binder or the use of carminic acid from Coccus cacti (an insect known as grana cochineal).

## 4. Conclusions

The employment of TEM-based techniques, more specifically 3D diffraction tomography coupled with ASTAR, allows for phase and orientation mapping and adds insight and hyperfine knowledge on studying artefacts and cultural heritage materials. The new information provided by the above instrumentation refers to crystallographic data (unit cell parameters, symmetry, and atomic positions) of unique nanocrystalline relics or secondary minerals that occur in the matrix of glasses, glazes, pigments, ceramics, etc.; additionally, it can thus distinguish between crystalline and amorphous areas of the samples having the advantage of the quasi-destructive approach due to minimal sample requirements (a few microns) the technique requires.

We should emphasize that in cultural heritage studies, the majority of artefacts, especially those characterized as pyrometallurgical products such as pottery, metals, glasses, and pigments as well as composite materials that underwent the heating process, incorporate several crystal phases where our detailed approach based on phase mapping at the nanoscale level can effectively identify, characterize, and thus separate uniquely amongst the crystalline phases; the possibility of the unique characterization of nanocrystals is based on the electron diffraction tomography (ADT) approach, which can then by further verified by detailed EDS data. The total approach of TEM examination, assisted by ED tomography, is superior to other conventional instrumentations operating at microscale (e.g., XRF, EDS, Raman, XRD, and EPMA) but also can be superior to most actual synchrotron single-crystal techniques, due to its limitation of crystal sizes of ca. 1-5 microns where in contrast with TEM-based crystallographic techniques, crystals of some tens of nm can effectively be examined.

The present study achieved the nanocrystalline mapping of pigments from acknowledged artefacts such as Greek glass amphorisks, Roman glass tesserae, and pre-Hispanic Maya pigments using TEM electron crystallography, thus enhancing our knowledge on the sophistication of the ancient color pallet; for example, the presence of white rutile TiO_2_ particles in Maya paintings has never been reported until recently [25] and could not be spotted by X-ray or other bulk techniques.

Additionally, phase and orientation mapping allows easily discriminating between amorphous and crystalline areas, which are not possible to recognize by TEM image contrast [9]. Identification of the nanoparticle phase and orientation, also in correlation with their amorphous matrix, may shed new light on the technological choices employed during the ancient kiln operation, reflected on the coloration, appearances, and mechanical properties of the final products.

A key issue on the studies of ancient artefacts is the complexity and heterogeneity of their matrixes, which is the driving force for research approaches at the single-crystal level, where even advanced techniques such as microfocused beam synchrotron techniques may lack a finer resolution posed by archaeology and the works of art.

In conclusion, the present study demonstrated that the combination of ADT and ASTAR mapping can provide the chemical and structural information required for the nanoscale description of ancient composite materials. ADT and ASTAR can be used with any TEM (100-300 kV) having sufficient angular tilt, precession electron diffraction hardware, and 3D electron diffraction tomography software.

## Figures and Tables

**Table 1:** EPMA microanalysis data performed on several areas of the fresh blue glass sample.

Oxide detection in wt%																		
No.	SiO_2_	Al_2_O_3_	MgO	Na_2_O	TiO_2_	CaO	SnO_2_	K_2_O	MnO	FeO	CoO	CuO	SO_3_	PbO	Cl	Ag_2_O	Sb_2_O_5_	Total
1	75.75	2.45	0.45	0.02	0.06	6.23	0	0.24	0.01	0.88	0.25	0.17	0.15	0.05	1.29	0.01	0.21	84.22
2	72.22	2.42	0.45	0.02	0.03	6.56	0	0.26	0.01	0.84	0.26	0.17	0.14	0	1.22	0	0.20	84.8
3	74.81	2.45	0.55	0.01	0.05	6.26	0	0.24	0.01	0.92	0.25	0.19	0.13	0	1.16	0	0.18	87.21
4	72.23	2.45	0.53	0.02	0.07	6.42	0	0.26	0.00	0.91	0.27	0.17	0.13	0	1.00	0	0.21	84.67
5	72.64	2.44	0.51	0.03	0.07	6.45	0	0.27	0.01	0.93	0.27	0.17	0.15	0.03	1.21	0.01	0.21	85.4
6	72.38	2.51	0.56	0.03	0.07	6.40	0	0.27	0.01	0.90	0.27	0.18	0.09	0.0	1.03	0	0.19	84.89
7	71.29	2.4	0.53	0.02	0.09	6.60	0	0.23	0.01	0.93	0.27	0.17	0.13	0.0	1.07	0	0.18	83.92

Analyzed areas are shown in Figure 5.

## Data Availability

The data used to support the findings of this study are available from the corresponding author upon request.
